# C30F12.4 influences oogenesis, fat metabolism, and lifespan in *C. elegans*

**DOI:** 10.1007/s13238-016-0308-z

**Published:** 2016-09-08

**Authors:** Lu Wang, Fei Xu, Guishuan Wang, Xiaorong Wang, Ajuan Liang, Hefeng Huang, Fei Sun

**Affiliations:** 1 International Peace Maternity & Child Health Hospital, Shanghai Key laboratory for Reproductive Medicine, School of Medicine, Institute of Embryo-Fetal Original Adult Disease, Shanghai Jiaotong University, Shanghai, 200030 China; 2 School of Life Sciences, University of Science and Technology of China, Hefei, 230026 China

**Keywords:** C30F12.4, oogenesis, fat metabolism, lifespan

## Abstract

Reproduction, fat metabolism, and longevity are intertwined regulatory axes; recent studies in *C. elegans* have provided evidence that these processes are directly coupled. However, the mechanisms by which they are coupled and the reproductive signals modulating fat metabolism and lifespan are poorly understood. Here, we find that an oogenesis-enriched gene, *c30f12.4*, is specifically expressed and located in germ cells and early embryos; when the gene is knocked out, oogenesis is disrupted and brood size is decreased. In addition to the reproductive phenotype, we find that the loss of *c30f12.4* alters fat metabolism, resulting in decreased fat storage and smaller lipid droplets. Meanwhile, *c30f12.4* mutant worms display a shortened lifespan. Our results highlight an important role for *c30f12.4* in regulating reproduction, fat homeostasis, and aging in *C. elegans*, which helps us to better understand the relationship between these processes.

**Electronic supplementary material:**

The online version of this article (doi:10.1007/s13238-016-0308-z) contains supplementary material, which is available to authorized users.

## Introduction

Reproduction, fat metabolism, and longevity are interconnected with each other, and numerous observations have suggested that reproduction can influence organismal lifespan and aging (Hansen et al., 2013). In many species, an abolished or reduced reproductive system can increase fat storage and lead to weight gain (Corona et al., 2009; Judd et al., 2011). In *C. elegans*, signals from the reproductive system can regulate longevity, and ablation of germ cells can alter fat metabolism and significantly prolong lifespan (Hsin and Kenyon, 1999; Goudeau et al., 2011). Recent studies have provided the most direct molecular evidence in *C. elegans* for the connection between reproduction, fat metabolism, and lifespan, showing that germline removal not only affects fat metabolism but also extends lifespan profoundly (Goudeau et al., 2011; McCormick et al., 2012; Wollam et al., 2012; Khanna et al., 2014).

Several pathways have been indicated to have important roles in coupling reproduction, fat metabolism, and life span. One of these is the insulin/IGF-1 signaling pathway: in *C. elegans*, one of the most important components of the insulin/IGF-1 signaling pathway is *daf-16*, a FOXO transcription factor that has an essential role in lifespan extension upon germline loss (Lin et al., 1997; Berman and Kenyon, 2006; Kenyon, 2010). Another is the steroid/NHR signaling pathway: this pathway includes many regulators, such as *daf-12*, a nuclear hormone receptor, and *daf-9*, a cytochrome P450 similar to CYP27A1. These two regulators are specific to germline signaling in their longevity-promoting effects because *daf-9* mutant worms display an extended lifespan, and loss-of-function mutations in *daf-12* increase lifespan in males (Gems et al., 1998; Jia et al., 2002; McCormick et al., 2012). Other reports also suggest that insulin/IGF-1 and steroid hormone/NHR signaling interact with each other to promote lifespan extension in germline-ablated worms (Berman and Kenyon, 2006).

Even though the existence of multiple links between reproduction, fat metabolism, and lifespan has been supported in so many works, these are still many intriguing questions to be addressed. In this study, we found that the protein, C30F12.4, from reproductive system and early embryo, could regulate fat homeostasis and lifespan in *C. elegans*. Our results define the physiological roles for C30F12.4 in regulating oogenesis, fat storage, the size of lipid droplets, and aging.

## Results

### Using dual sgRNAs to knock out *c30f12.4*

C30F12.4, which has been reported to be a strictly maternal gene, is one of the oogenesis-enriched genes of heretofore unknown function (Spencer et al., 2011). RNAi inactivation of *c30f12.4* resulted in significantly decreased brood size in hermaphrodites (Fig. S1), prompting us to study the function of *c30f12.4* in regulating worm development. To further characterize *c30f12.4*, we sought to generate null alleles using CRISPR/Cas9 technology with dual sgRNAs.

**Figure 1:**
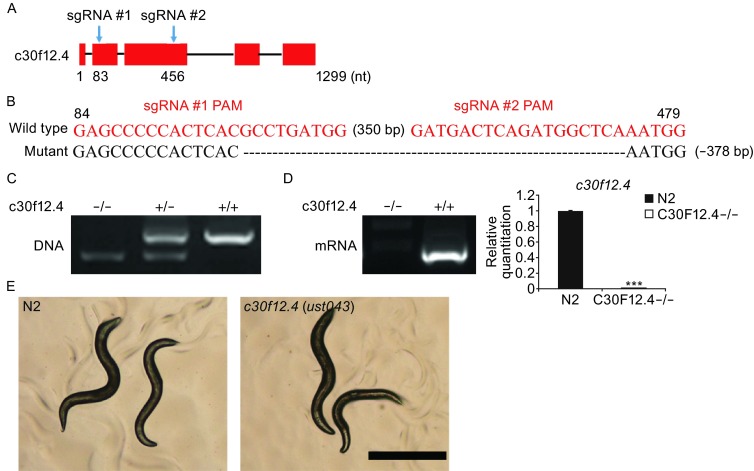
**Dual sgRNA-guided deletion of the** ***c30f12.4*** **gene**. (A) Schematic of the *c30f12.4* gene. Positions of sgRNA-guided cleavage sites are indicated. (B) Sequence alignments of the *c30f12.4* gene in wild-type and mutant worms. The sgRNA PAM sequence is labeled in red. The number of deleted (-) bases is shown to the right of each indel. The numbers in parentheses within the sequence represent the number of bases not shown. Numbers on the top of sequences indicate positions relative to the transcription start site. (C) PCR amplification of the targeted region in N2 and *c30f12.4* deletion mutant. (D) PCR amplification and real time PCR analysis of *c30f12.4* gene expression in total RNAs from the N2 and *c30f12.4* mutant. ***P < 0.001, *t*-test. (E) The phenotype between N2 and *c30f12.4* mutant. Scale bar = 0.5 mm

We coinjected sgRNAs targeting exons 2 and 3 of *c30f12.4*, Cas9 and mCherry expression plasmids into young adult N2 worms (Fig. 1A). F1 animals expressing mCherry were first transferred to NGM plates. After three days, F1 with corresponding F2 progeny were harvested and screened by PCR amplification. We identified lesions in the *c30f12.4* gene consistent with Cas9-directed cleavage (Fig. 1B). This deletion was further confirmed by PCR amplification of DNA, mRNA expression, and DNA sequencing (Fig. 1B–D). The large deletion may reflect a simultaneous cleavage directed by the two sgRNAs, whose targets are separated by 378 bases in this experiment. Nevertheless, we found no difference in movement, appearance, and growing rate in the first three days post hatch between N2 and *c30f12.4* mutant animals (Fig. 1E).

### Disruption of *c30f12.4* causes female sterility in worms

**Figure 2:**
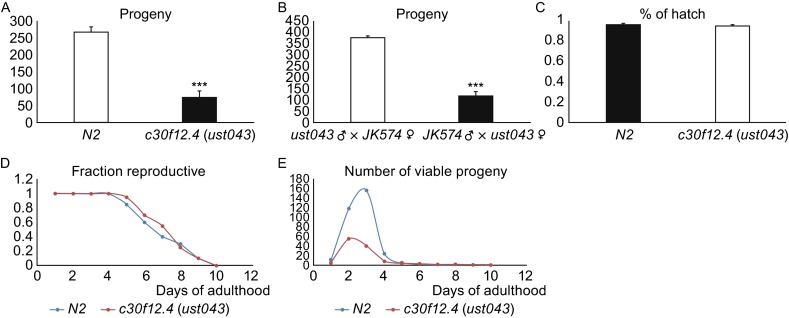
**Disruption of** ***c30f12.4*** **causes female sterility in worms**. (A) Brood size of wild-type or mutant worms was determined at 20°C. The x-axis indicates corresponding genotypes; the y axis is the mean value of total number of progeny per animal. Greater than six animals per trial, average of three trials. ***P < 0.001, *t*-test. (B) The number of progeny after mating between *fog-2* (*JK574*) and *c30f12.4* (*ust043*). Four animals per trial, average of three trials. ***P < 0.001, *t*-test. (C) Percentage of embryos hatched after laying from day two wild-type and *c30f12.4* adults. Greater than 500 eggs per trial, average of three trials. (D) Reproductive span of wild-type and *c30f12.4.* Ten animals per trial, average of three trials. (E) *C30f12.4* mutants produce fewer early and total progeny than wild type. Ten animals per trial, average of three trials

**Figure 3:**
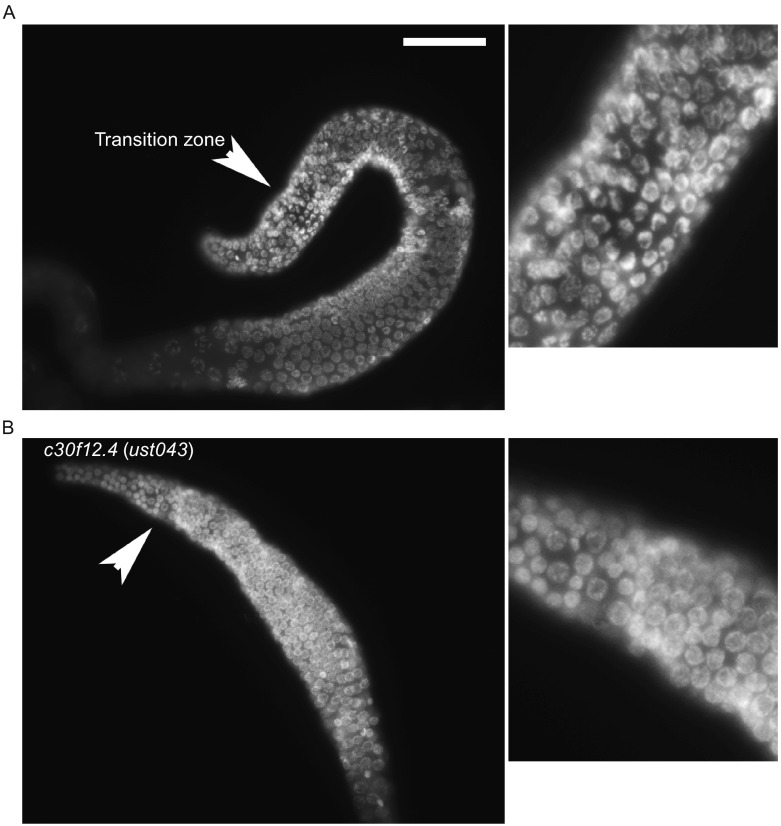
**Absence of transition zone in** ***c30f12.4*** **mutant worms**. Each panel shows Hoechst-stained nuclei in an isolated gonad. (A) Wild-type gonad, arrowhead: transition zone. (B) *C30f12.4* mutant gonad. Scale bar = 50 μm

As mentioned above, treating animals with RNAi to reduce *c30f12.4* levels resulted in decreased brood size in worms (Fig. S1). We therefore first examined the progeny of the *c30f12.4* (*ust043*), which exhibited a 3-fold decrease in brood size at 20°C in hermaphrodites (Fig. 2A). Moreover, when we crossed the male and female *c30f12.4* (*ust043*) and *fog-2* (*JK574*) worms, we found that the progeny of the *JK574* male × *ust043* female cross exhibited a 3-fold decrease relative to the *JK574* female × *ust043* male cross (Fig. 2B), indicating that knockout of *c30f12.4* may only influence oogenesis, but not spermatogenesis. In order to confirm that a dysfunction in oogenesis rather than hatch led to decreased brood size, we examined the hatch rate in mutant and wild-type worms. As expected, the hatch rates of *c30f12.4* mutants were comparable to those of the wild type (Fig. 2C). Meanwhile, the onset of progeny production in *c30f12.4* mutants was not delayed and progeny was steadily produced at the age when reproduction ceased in the wild type (Fig. 2D and 2E). The overall difference was that *c30f12.4* mutants produced fewer early and total progeny over the same period of time (Fig. 2E). During meiosis, a major spatial reorganization of chromosomes within nuclei occurs in the transition zone region of the germ line, corresponding to the leptotene/zygotene stages of meiotic prophase (Dernburg et al., 1998). The chromatin becomes asymmetrically localized and concentrated toward one side of the nucleus, generating polarity that imparts a distinctive crescent-shaped appearance to the Hoechst-stained chromatin in transition zone nuclei that is readily evident, even in low magnification images (Fig. 3A). Nonetheless, in the *c30f12.4* germ line, we could not find any crescent-shaped nuclei in any region of the gonad (Fig. 3B).

### Loss of *c30f12.4* alters lipid metabolism

**Figure 4:**
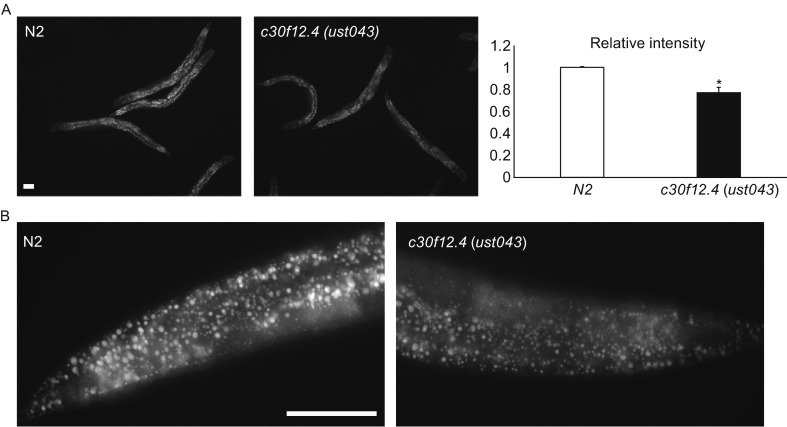
**Loss of** ***c30f12.4*** **altered fat storage in** ***C. elegans***. Late L4 stage worms were fixed with 40% isopropanol and stained with Nile Red. Images were captured using identical settings and exposure time for each image. Loss of *c30f12.4* lead to decreased fat storage (A) and smaller lipid droplets (B). *P < 0.05, *t*-test. Scale bar = 50 μm

**Figure 5:**
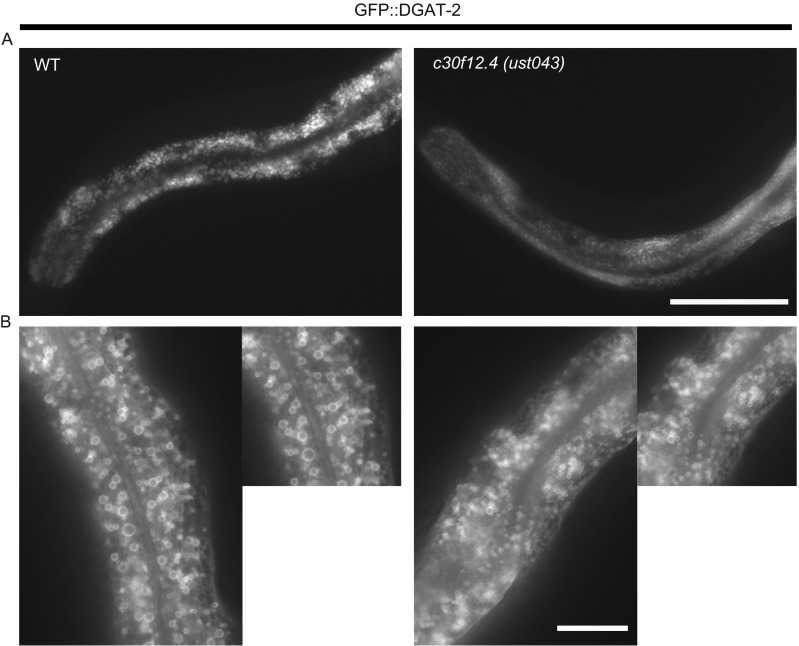
**Loss of** ***c30f12.4*** **altered fat storage in** ***hjSi56*** **worms**. L4 stage wild-type and *c30f12.4* mutant worms on an *hjSi56* background were detected directly. Images were captured using identical settings and exposure time for each image. Loss of *c30f12.4* led to decreased fat storage (A) and smaller lipid droplets (B). Scale bar = 20 μm (A) and 50 μm (B)

**Figure 6:**
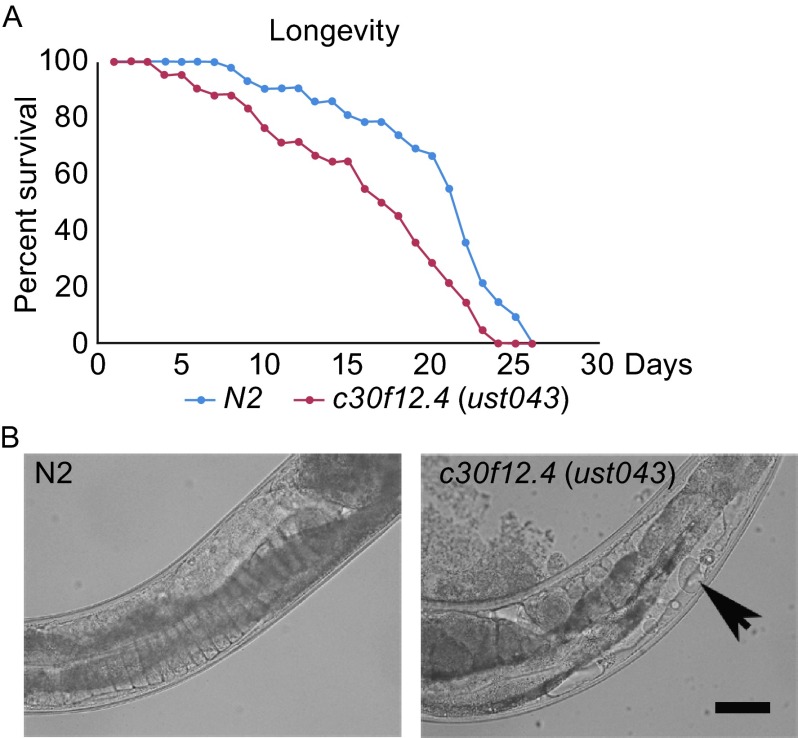
***c30f12.4*** **(** ***ust043*** **) worms have reduced life span**. (A) Adult life span of wild-type (blue line) and *c30f12.4* (*ust043*) (red line) worms. Sixty worms per trial, average of three trials. (B) Vacuole-like structures (black arrowhead), appear in *c30f12.4* (*ust043*) worm bodies towards the end of the reproductive period. Scale bar = 50 μm

Besides the function of c30f12.4 in influencing oogenesis in worms, we also found that the gene could affect fat storage. We compared the fat content between the wild-type and *c30f12.4* mutant worms by fixed Nile Red staining when they were fed with the standard *Escherichia coli* OP50 diet *ad libitum*. Mutation of *c30f12.4* led to a significant, 23% reduction of intracellular lipids compared with wild-type worms (Fig. 4A). Meanwhile, loss of *c30f12.4* resulted in a smaller size of lipid droplets (Fig. 4B). In order to further confirm the role of *c30f12.4* in regulating fat homeostasis in living worms, we crossed *c30f12.4* mutant worms with *hjSi56*, which can express GFP-DGAT-2, a marker of lipid droplets (Xu et al., 2012; Klemm et al., 2013). In wild-type worms, the lipid droplets are clear, bright, and relatively large, while in mutant worms, they are blurry and smaller (Fig. 5A and 5B). Moreover, we found that the expression of *fat-5* and *fat-7* were decreased in mutant worms (Fig. S2). These results indeed demonstrated that *c30f12.4* could affect fat homeostasis. Next, we also asked whether mutation of *c30f12.4* could influence life span because of its role in regulating fat metabolism. We found that *c30f12.4* mutant worms displayed significantly reduced animal survival (Fig. 6A). Moreover, we found that vacuole-like structures appeared in mutant worm bodies towards the end of the reproductive period from the seventh day of adulthood, which indicates an aging worm (Herndon et al., 2002), while in normal worms, there are no such structures (Fig. 6B). The phenotype is associated with the decreased life span we detected in mutant worms. These results indicated that the disrupted fat homeostasis in *c30f12.4* mutant worms may lead to aging and a shortened life span.

### C30F12.4 are specifically expressed in germ cells and early embryos

**Figure 7:**
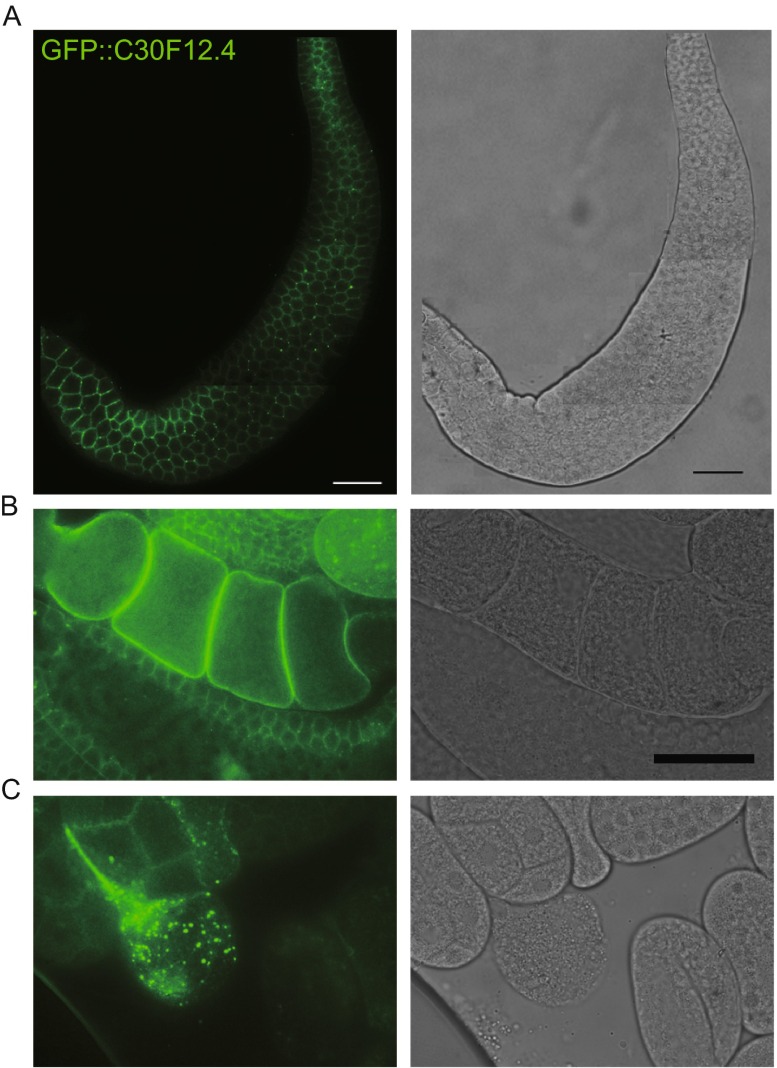
**GFP::C30F12.4 was specifically expressed and located in germ cells and early embryos**. (A and B) C30F12.4 was mainly located in the cytoplasm around the germ cells and everywhere in oocytes. (C) C30F12.4 displayed a punctate signal in single-cell embryos. Scale bar = 20 μm

To detect the endogenously produced protein encoded by *c30f12.4* and the location of C30F12.4 in worms, we constructed a 3×FLAG-GFP:C30F12.4 transgene worm using the Mos1-mediated single-copy insertion (mosSCI) system. We found that the fused GFP::C30F12.4 was expressed very well and could be detected clearly under the green fluorescent protein channel. C30F12.4 was expressed and located specifically in germ cells and early embryos, especially in oocytes and single-cell embryos in worms (Fig. S3). Moreover, this transgene can rescue the decreased brood size of *c30f12.4* mutant worms from 83 to 227 (Fig. S4). In order to further identify the localization of C30F12.4 in germ cells and embryos, we isolated gonads and embryos and found that C30F12.4 was mainly located in the cytoplasm around the germ cells and everywhere in oocytes (Fig. 7C). Meanwhile, we found that C30F12.4 displayed a punctate signal in single-cell embryos, and the expression of C30F12.4 was decreased gradually during embryo development (Fig. 7D).

## Discussion

In this paper, we have identified *C. elegans* C30F12.4 as the special regulator that links reproduction, fat metabolism, and lifespan. C30F12.4 was reported as an oogenesis-enriched gene, and we found that it was indeed expressed and located in germ cells and early embryos. When this gene was knocked out, oogenesis was disrupted with a missed transition zone, leading to decreased brood size. Meanwhile, fat metabolism in the mutant worms was also influenced: in mutant worms, fat storage was decreased and lipid droplets became smaller, concomitant with a shortened lifespan.

As reported in many papers, reproduction, fat metabolism, and lifespan are interconnected. Germline signals can modulate the activity of an insulin/IGF-1 (insulin-like growth factor) pathway, which has an important role in regulating aging. If the cells that give rise to the germ line are killed or germline stem cells (GSC) undergo cell cycle arrest, the lifespan of the worms is extended (Hsin and Kenyon, 1999; Wang et al., 2008). However, no paper has reported that a protein specifically expressed in the germline can influence not only oogenesis but also fat metabolism and lifespan. C30F12.4 is expressed mainly in oocytes and single-cell embryos, while its function couples oogenesis, fat homeostasis, and life span. Besides the phenotype we have found in *c30f12.4* mutant worms, we also determined, using IP and MS analysis, which C30F12.4 could interact with many proteins (Fig. S5 and Table S2). Some of these proteins, such as SET-30, SRW-100, and C27B7.7, have been reported to have an important role in regulating lifespan by RNAi (Hamilton et al., 2005; Ni et al., 2012). Other proteins, like SMC-3, RPL-22, EGO-1, and so on, play some special roles in germline development (Claycomb et al., 2009; Kalis et al., 2010; Green et al., 2011), indicating that C30F12.4 may regulate oogenesis, fat metabolism, and lifespan by interacting with other proteins. However, the specific mechanism of C30F12.4 in regulating these processes and how this protein cooperates with other proteins to link these processes needs further study.

## Materials and methods

### Strains

The wild-type strain was Bristol N2. All animals were raised at 20°C. The following alleles and transgenes were used: shg372: *c30f12.4* (ust043), *fog-2* (JK574), hjSi56 [vha-6p::3×FLAG-TEV-GFP::dgat-2::let-858 3′UTR] IV, allele: ustIS039 strain: shg401 (pc30f12.4::3×FLAG-GFP::c30f12.4::c30f12.4 3′UTR).

### RNAi

RNAi experiments were performed as described previously (Chen et al., 2014).

### Construction of sgRNA expression plasmids

We manually searched for target sequences consisting of G(N)_19_ NGG for *c30f12.4* near the desired mutation sites. The unc-119 target sequence in the pU6::unc-119 sgRNA vector was replaced with the desired target sequence (Wiedenheft et al., 2012; Friedland et al., 2013; Chen et al., 2014). The primer sequences used for the construction of sgRNA expression plasmids are listed in Table S1.

### Micro-injection

DNA mixtures were micro-injected into the gonads of young adult N2 *C. elegans*. For plasmids used in dual sgRNA experiments, we injected 50 ng/mL Cas9 expressing vector, 50 ng/mL sgRNA #1, 50 ng/mL sgRNA #2, and 5 ng/mL pCFJ90 vector.

### Screening for deletion mutants by PCR

After injection, F1 worms which expressed mCherry were transferred to individual NGM plates. After 3 days, F1 with corresponding F2 progeny were harvested and total DNA was extracted and screened by PCR amplification with primers outside of the sgRNA-targeted regions. Mutant worms containing the deletion were singled to NGM plates and then confirmed by PCR amplification and DNA sequencing. The mutant worm strain was named shg372: *c30f12.4* (ust043). The primers used for PCR screening are listed in Table S1.

### 3×FLAG-GFP:C30F12.4 transgene insertion by Mos1-mediated single-copy insertion (mosSCI) system

MosSCI was performed as previously described (Frokjaer-Jensen et al., 2014). Briefly, the pc30f12.4::3×FLAG-GFP::c30f12.4::c30f12.4 3′UTR construct was cloned into PCFJ151 (PCFJ151-C30F12.4) and DNA mix (PCFJ151-C30F12.4 50 ng/μL, PGH8 5 ng/μL, PCFJ90 2.5 ng/μL, PCFJ104 5 ng/μL, PJL43.1 50 ng/μL, Peel-1 10 ng/μL) were coinjected into the young adult strain EG4322 seeded with HT115. After injection, five worms were placed on each NGM plate and incubated at 25°C until starvation. Then, animals were heat-shocked for 2 h at 34°C in an air incubator, and the day after, the worm that was alive and moved well but lacked the fluorescent co-injection markers was singled. After five days, it was verified that all of the offspring moved well and identification was performed by PCR and Western blot (Fig. S6). The GFP::C30F12.4 transgene worm strain was named allele: ustIS039 strain: shg401 (pc30f12.4::3×FLAG-GFP::c30f12.4::c30f12.4 3′UTR). The primer sequences used for the construction of PCFJ151-C30F12.4 are listed in Table S1.

### Progeny production analysis

Individual synchronized L4 hermaphrodites were moved to fresh plates and the number of progeny produced by each individual was counted daily until reproduction ceased for at least two days. All experiments were performed in three independent replicates at 20°C with at least five individuals per strain, once.

### Hatching rate

Eggs were synchronized and allowed to develop at 20°C until day two of adulthood. Ten synchronized hermaphrodites were transferred to a new plate and allowed to lay eggs for 6 h, then eggs and young adult progeny were counted. All experiments were performed in three independent replicates.

### Reproductive span analysis

Individual synchronized L4 hermaphrodites were moved to fresh plates daily until reproduction ceased for at least two days. The last day of viable progeny production was noted as the day of reproduction cessation for each individual. All experiments were performed in three independent replicates at 20°C with at least 10 individuals per strain, once.

### Lifespan analysis of *C. elegans*

Lifespan analysis was carried out at 20°C with worms maintained for several generations at 20°C on consistent dietary *Escherichia coli* OP50 diet *ad libitum*. L4 worms were transferred to fresh plates at a density of 10 worms per plate at the beginning of the experiment, day zero (Kenyon et al., 1993). No FUdR or antibiotics were included in the plates. Worms were transferred to fresh plates daily until they stopped laying eggs, after which they were transferred every three days. Animals were considered dead when they no longer responded to a gentle tap with a worm pick. The mean and maximum lifespans were determined by the average of three independent trials, each using 60 animals.

### Nile Red staining

Nile Red staining was performed as previously described (Pino et al., 2013; Pang et al., 2014). Briefly, synchronized late L4 worms of the indicated genotypes were collected, washed with M9 buffer, and fixed in 40% isopropanol at room temperature for 3 min and stained in 3 mg/mL Nile Red (Sigma) working solution in the dark for 2 h. Worms were then washed with M9 for 10 min in the dark, three times, mounted onto slides and imaged under the green fluorescent protein channel. All experiments were performed in three independent replicates with at least 20 individuals per strain, once.

### Immunoprecipitation assay and mass spectrometry

Immunoprecipitation (IP) was performed as described previously (Wang et al., 2014) and mass spectrometry (MS) analysis was performed by Shanghai applied protein technology Co. Ltd.

## Electronic supplementary material

Below is the link to the electronic supplementary material.

Supplementary material 1 (PDF 986 kb)
