# Supplementary material for: C30F12.4 influences oogenesis, fat metabolism, and lifespan in C. elegans
Source: Protein Cell. 2016 Sep 8;7(10):714–21. doi: 10.1007/s13238-016-0308-z (PMC5055490; doi:10.1007/s13238-016-0308-z)

C30F12.4 influences oogenesis, fat metabolism and lifespan in *C.elegans*

Lu Wang, Fei Xu, Guishuang Wang, Xiaorong Wang, Ajuan Liang, Hefeng Huang and Fei Sun

Figure S1: Brood size of wile type and *c30f12.4* RNAi worms. All experiments were performed for three independent trials with at least 5 individuals per strain, once. *p<0.05, *t*-test.

Figure S2: Real-time PCR analysis of *fat-5* and *fat-7* gene expression in total RNAs from late L4 stage of N2 and *c30f12.4* mutant worms. *P < 0.05, *t*-test.

Figure S3: GFP::C30F12.4 was specifically expressed and located in germ cells and early embryos. Scale bar = 20 μm.

Figure S4: Brood size of wild-type, *c30f12.4* (ust 043) and *c30f12.4; c30f12.4::gfp* worms. Greater than five animals per trial, average of three trials. ****p* < 0.001, *t*-test.

Figure S5: Co-immunoprecipitation (IP) using antibodies to GFP, followed by commassie blue staining and Western blotting using an antibody to GFP in GFP::C30F12.4 and N2 worms.

Figure S6: Identification of GFP::C30F12.4 transgene worms by PCR (A) and Western blotting (B).

Table S1 List of primers

Gene	Sequence (5' to 3')
sgRNA#1	Forward: GAGCCCCCACTCACGCCTGAGTTTTAGAGCTAGAAATAGC
	Reverse: TCAGGCGTGAGTGGGGGCTCAAACATTTAGATTTGCAATT
sgRNA#2	Forward: GATGACTCAGATGGCTCAAAGTTTTAGAGCTAGAAATAGC
	Reverse: TTTGAGCCATCTGAGTCATCAAACATTTAGATTTGCAATT
c30f12.4 (mutation)	Forward: GCTCAAGATGACAATTAATCCGACT
	Reverse: GACTAACCTTTCTCAATCGATCTTG
p*c30f12.4*	Forward: CGTGGATCCAGATATCCTGCAGGTTAGTTATAAAAGCTTCGT
	Reverse: ATCAGTCGGATTAATTGTCATCTTGAGCAACGATTTTCCGA
3×FLAG-GFP	Forward: ATGACAATTAATCCGACTGATATGGACTACAAAGACCATGA
	Reverse: TCAGTCGGATTAATTGTCATTTTGTATAGTTCATCCATGC
c30f12.4 + 3'UTR	Forward: ATGACAATTAATCCGACTGA
	Reverse: TATAAGATCTAAAAATAATTCAAGAAAGAAAC

Table S2 Proteins from ME analysis which interacted with C30F12.4

Group number	Protein name
1	Uncharacterized protein OS=Caenorhabditis elegans GN=CELE_T09B4.5 PE=4 SV=1
2	Uncharacterized protein OS=Caenorhabditis elegans GN=hpo-10 PE=4 SV=1
3	INositol Polyphosphate-5-Phosphatase OS=Caenorhabditis elegans GN=inpp-1 PE=4 SV=1
4	Serpentine Receptor, class T OS=Caenorhabditis elegans GN=srt-47 PE=4 SV=2
5	Structural maintenance of chromosomes protein 3 OS=Caenorhabditis elegans GN=smc-3 PE=1 SV=1
6	AEX-2 related neuropeptide Receptor OS=Caenorhabditis elegans GN=aexr-3 PE=4 SV=1
7	Uncharacterized protein OS=Caenorhabditis elegans GN=CELE_F49H6.5 PE=4 SV=1
8	Uncharacterized protein OS=Caenorhabditis elegans GN=CELE_F32B4.4 PE=1 SV=3
9	60S ribosomal protein L22 OS=Caenorhabditis elegans GN=rpl-22 PE=1 SV=3
10	Uncharacterized protein OS=Caenorhabditis elegans GN=CELE_Y116A8A.150 PE=4 SV=1
11	Uncharacterized protein OS=Caenorhabditis elegans GN=CELE_H28G03.2 PE=4 SV=1
12	SCP-Like extracellular protein OS=Caenorhabditis elegans GN=scl-24 PE=4 SV=1
13	WD repeat-containing protein wdr-5.3 OS=Caenorhabditis elegans GN=wdr-5.3 PE=3 SV=1
14	Uncharacterized protein OS=Caenorhabditis elegans GN=CELE_T28D6.4 PE=4 SV=1
15	Enhancer of Glp-One (Glp-1) OS=Caenorhabditis elegans GN=ego-1 PE=2 SV=1
16	DAF-16/FOXO Controlled, germline Tumor affecting OS=Caenorhabditis elegans GN=dct-14 PE=4 SV=2
17	Uncharacterized protein OS=Caenorhabditis elegans GN=CELE_W04E12.7 PE=4 SV=1
18	Protein-tyrosine-phosphatase OS=Caenorhabditis elegans GN=CELE_W03F11.4 PE=4 SV=3
19	ATP synthase lipid-binding protein, mitochondrial OS=Caenorhabditis elegans GN=Y82E9BR.3 PE=3 SV=1
20	Uncharacterized protein OS=Caenorhabditis elegans GN=C55C3.6 PE=4 SV=1
21	CYtochrome P450 family OS=Caenorhabditis elegans GN=cyp-33d1 PE=3 SV=1
22	Uncharacterized protein OS=Caenorhabditis elegans GN=CELE_F12F6.1 PE=4 SV=1
23	Uncharacterized protein OS=Caenorhabditis elegans GN=CELE_ZC373.4 PE=4 SV=1
24	Tubulin alpha-8 chain OS=Caenorhabditis elegans GN=tba-8 PE=3 SV=2
25	Uncharacterized protein OS=Caenorhabditis elegans GN=CELE_R03E9.2 PE=4 SV=3
26	Ankyrin repeat and KH domain-containing protein R11A8.7 OS=Caenorhabditis elegans GN=R11A8.7 PE=3 SV=3
27	Uncharacterized protein OS=Caenorhabditis elegans GN=CELE_F44A2.3 PE=4 SV=5

	28	Uncharacterized protein OS=Caenorhabditis elegans GN=CELE_F15E6.6 PE=4 SV=3
	29	Serpentine Receptor, class W OS=Caenorhabditis elegans GN=srw-100 PE=4 SV=1
	30	QUAhog (Hedgehog related) OS=Caenorhabditis elegans GN=qua-1 PE=4 SV=1
	31	SET (Trithorax/polycomb) domain containing OS=Caenorhabditis elegans GN=set-30 PE=4 SV=3
	32	Uncharacterized protein OS=Caenorhabditis elegans GN=CELE_Y69A2AR.18 PE=3 SV=1
	33	Ig-like and fibronectin type-III domain-containing protein C27B7.7 OS=Caenorhabditis elegans GN=C27B7.7 PE=1 SV=2
	34	Uncharacterized protein OS=Caenorhabditis elegans GN=CELE_ZC15.3 PE=4 SV=1
	35	Uncharacterized protein OS=Caenorhabditis elegans GN=CELE_Y57G11B.1 PE=4 SV=1
	36	Auxilin OS=Caenorhabditis elegans GN=dnj-25 PE=2 SV=1
	37	Uncharacterized protein OS=Caenorhabditis elegans GN=CELE_F22B3.5 PE=4 SV=4
	38	Seven TM Receptor OS=Caenorhabditis elegans GN=str-12 PE=4 SV=2
	39	Peptidyl-prolyl cis-trans isomerase 4 OS=Caenorhabditis elegans GN=cyn-4 PE=1 SV=3
	40	Uncharacterized protein OS=Caenorhabditis elegans GN=CELE_Y39G10AR.11 PE=4 SV=1
	41	Ribosomal Protein, Large subunit OS=Caenorhabditis elegans GN=rpl-30 PE=1 SV=2
	42	Mitogen-activated protein kinase kinase kinase kinase OS=Caenorhabditis elegans GN=gck-2 PE=4 SV=1
	43	Ran GTPase-activating protein 2 OS=Caenorhabditis elegans GN=ran-2 PE=4 SV=3
	44	Coiled-coil and C2 domain-containing protein 1-like OS=Caenorhabditis elegans GN=Y37H9A.3 PE=3 SV=2
	45	F-box A protein OS=Caenorhabditis elegans GN=fbxa-210 PE=4 SV=1
	46	ACTin OS=Caenorhabditis elegans GN=act-5 PE=1 SV=1
	47	Nose resistant to fluoxetine protein 6 OS=Caenorhabditis elegans GN=nrf-6 PE=1 SV=3
	48	MAM (Meprin, A5-protein, PTPmu) domain protein OS=Caenorhabditis elegans GN=mam-3 PE=4 SV=3
	49	Seven TM Receptor OS=Caenorhabditis elegans GN=str-119 PE=4 SV=1
	50	Formin-homology and zinc finger domains protein 1 OS=Caenorhabditis elegans GN=fozi-1 PE=3 SV=1
	51	Na/Ca eXchangers OS=Caenorhabditis elegans GN=ncx-9 PE=4 SV=1
	52	Heavy Metal Tolerance factor OS=Caenorhabditis elegans GN=hmt-1 PE=2 SV=1
	53	Uncharacterized protein OS=Caenorhabditis elegans GN=CELE_T23B3.1 PE=4 SV=1
	54	Cytosolic carboxypeptidase 6 OS=Caenorhabditis elegans GN=ccpp-6 PE=2 SV=2
	55	Seven TM Receptor OS=Caenorhabditis elegans GN=str-156 PE=4 SV=2
	56	Uncharacterized protein OS=Caenorhabditis elegans GN=CELE_F01F1.3 PE=4 SV=2
	57	SMAll OS=Caenorhabditis elegans GN=sma-10 PE=4 SV=1

58	ATP synthase subunit beta, mitochondrial OS=Caenorhabditis elegans GN=atp-2 PE=1 SV=2
59	Uncharacterized protein OS=Caenorhabditis elegans GN=CELE_F19B2.7 PE=4 SV=4
60	Nuclear hormone receptor family member nhr-19 OS=Caenorhabditis elegans GN=nhr-19 PE=1 SV=2
61	Uncharacterized protein OS=Caenorhabditis elegans GN=C41G7.3 PE=4 SV=2
62	TWiK family of potassium channels OS=Caenorhabditis elegans GN=twk-29 PE=3 SV=2
63	Uncharacterized protein OS=Caenorhabditis elegans GN=C44H4.4 PE=4 SV=1
64	Protein PAT1 homolog 1 OS=Caenorhabditis elegans GN=patr-1 PE=3 SV=2
65	Homeobox protein cut-like ceh-44 OS=Caenorhabditis elegans GN=ceh-44 PE=3 SV=1
66	Uncharacterized protein OS=Caenorhabditis elegans GN=CELE_ZC376.3 PE=4 SV=1
67	Protein nipi-3 OS=Caenorhabditis elegans GN=nipi-3 PE=1 SV=1
68	Ectonucleotide pyrophosphatase/phosphodiesterase C27A7.1 OS=Caenorhabditis elegans GN=C27A7.1 PE=1 SV=1
69	Uncharacterized protein OS=Caenorhabditis elegans GN=C01B4.8 PE=4 SV=1
70	Serpentine receptor class alpha-18 OS=Caenorhabditis elegans GN=sra-18 PE=3 SV=1
71	F-box C protein OS=Caenorhabditis elegans GN=fbxc-22 PE=4 SV=2
72	BTB (Broad/complex/Tramtrack/Bric a brac) domain protein OS=Caenorhabditis elegans GN=btb-16 PE=4 SV=3
73	Innexin-6 OS=Caenorhabditis elegans GN=inx-6 PE=2 SV=1
74	Uncharacterized protein OS=Caenorhabditis elegans GN=CELE_F36A4.2 PE=4 SV=1

Fig. S1

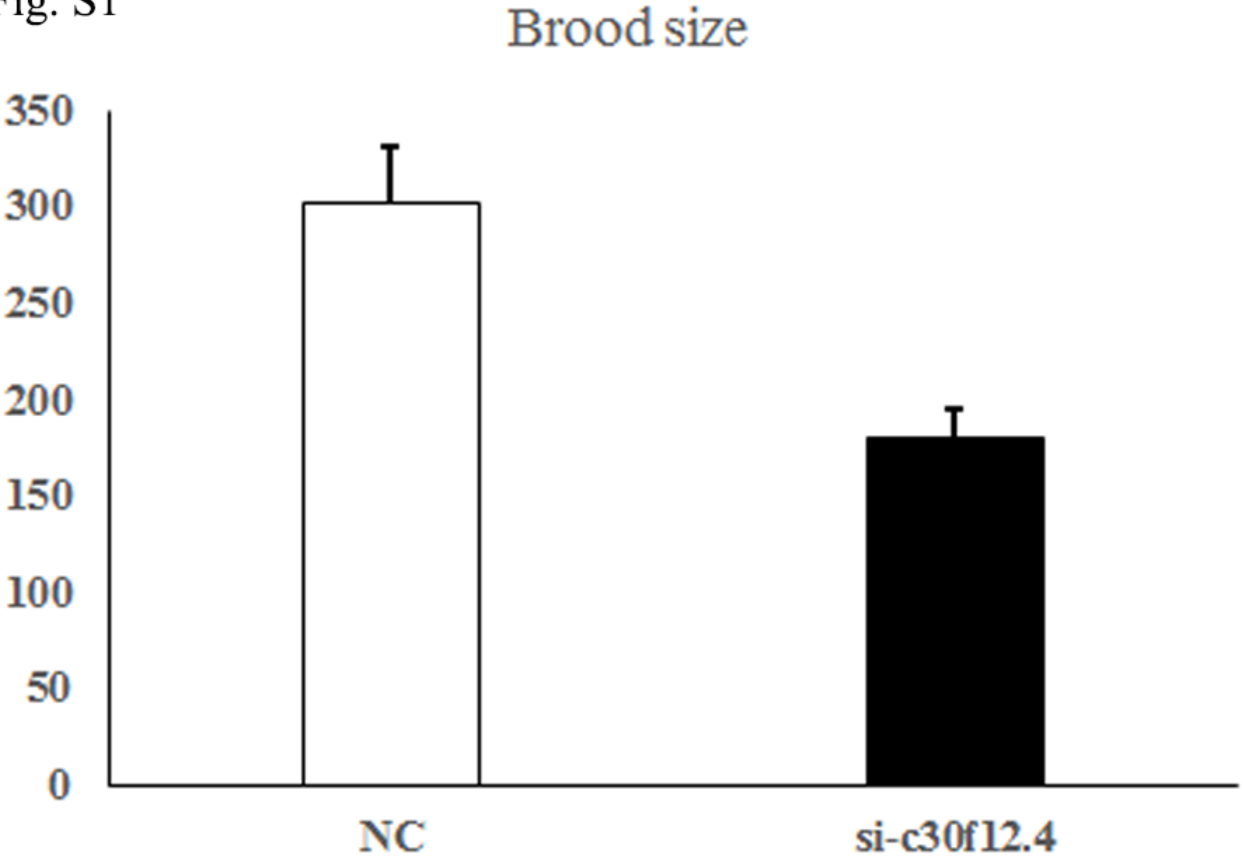

Fig. S2

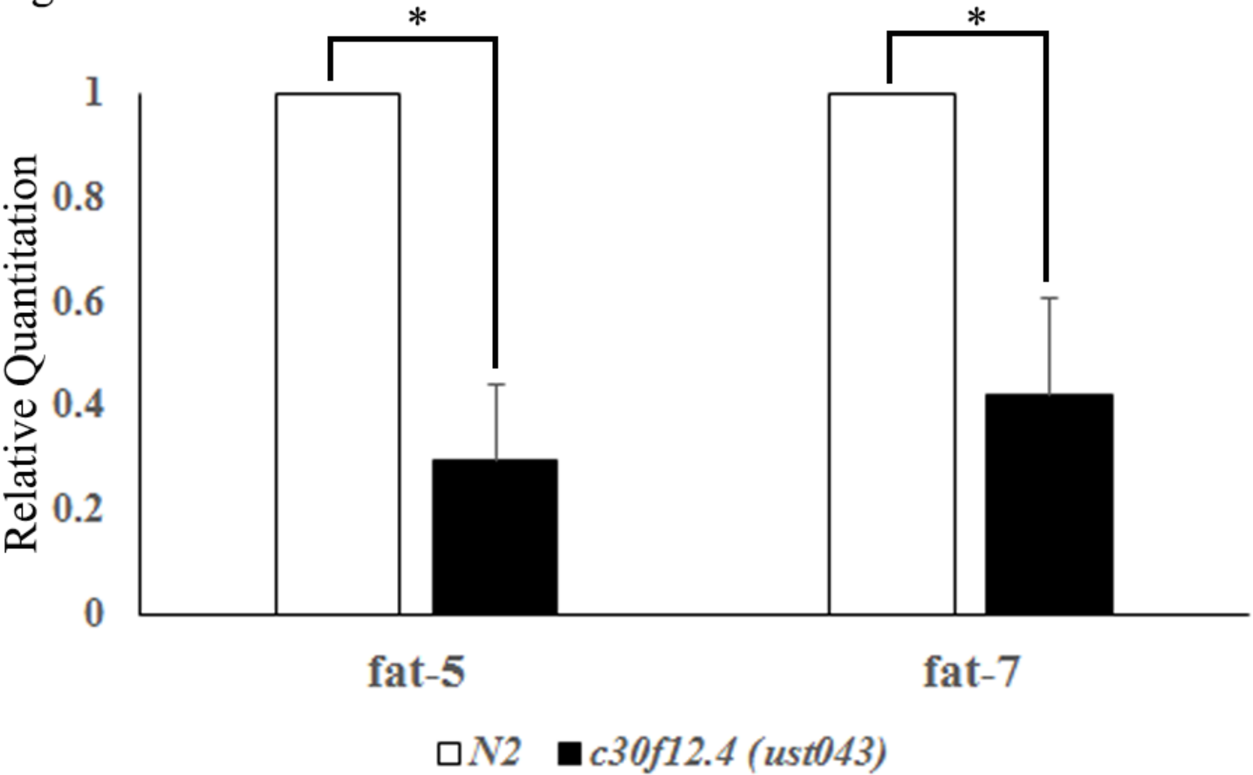
*
*
Relative Quantitation
1
0.8
0.6
0.4
0.2
0
fat-5
fat-7
N2
c30f12.4 (ust043)

Fig .S3

A

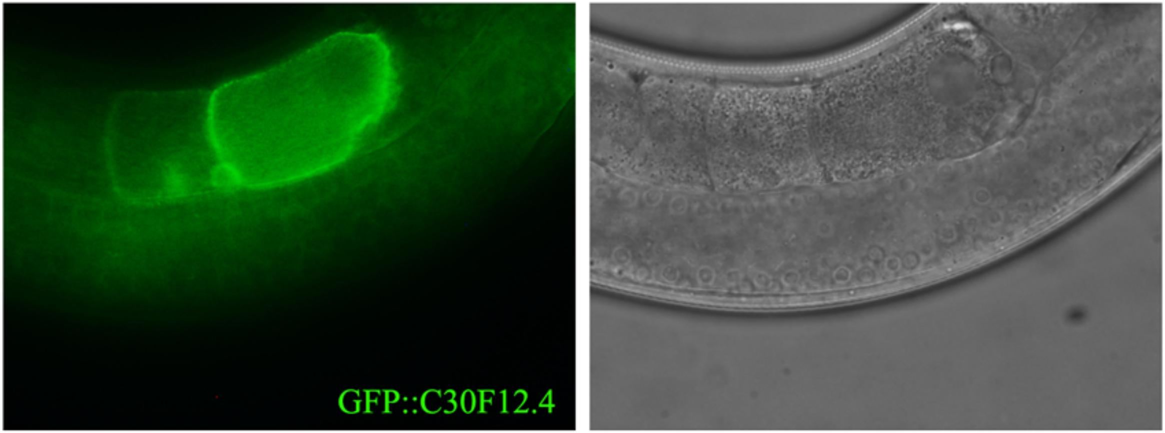

B

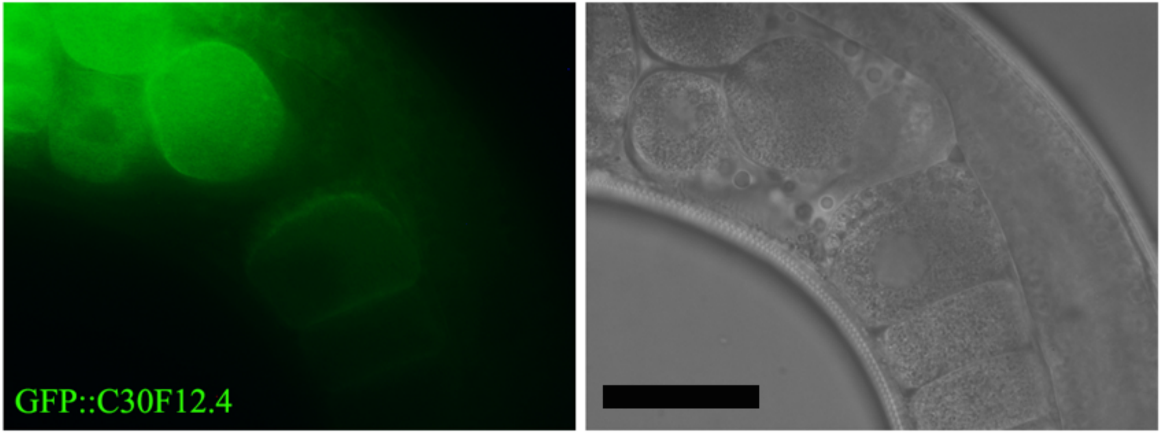

Fig. S4

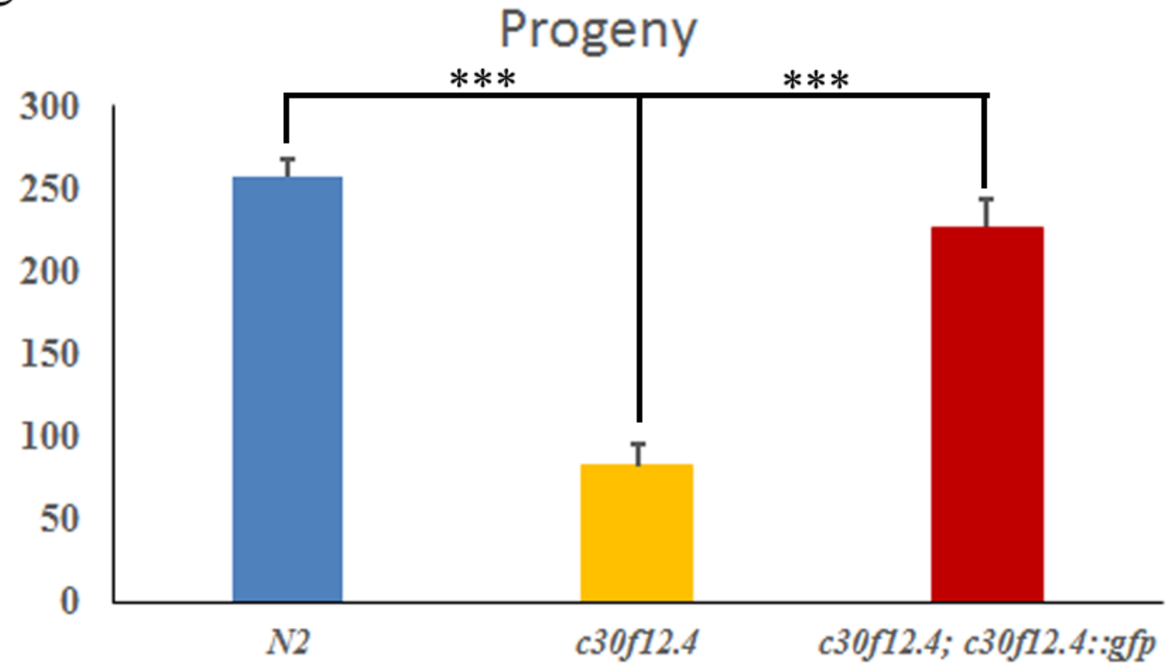

Progeny
***
***
300
250
200
150
100
50
0
N2
c30f12.4
c30f12.4; c30f12.4::gfp

Fig. S5

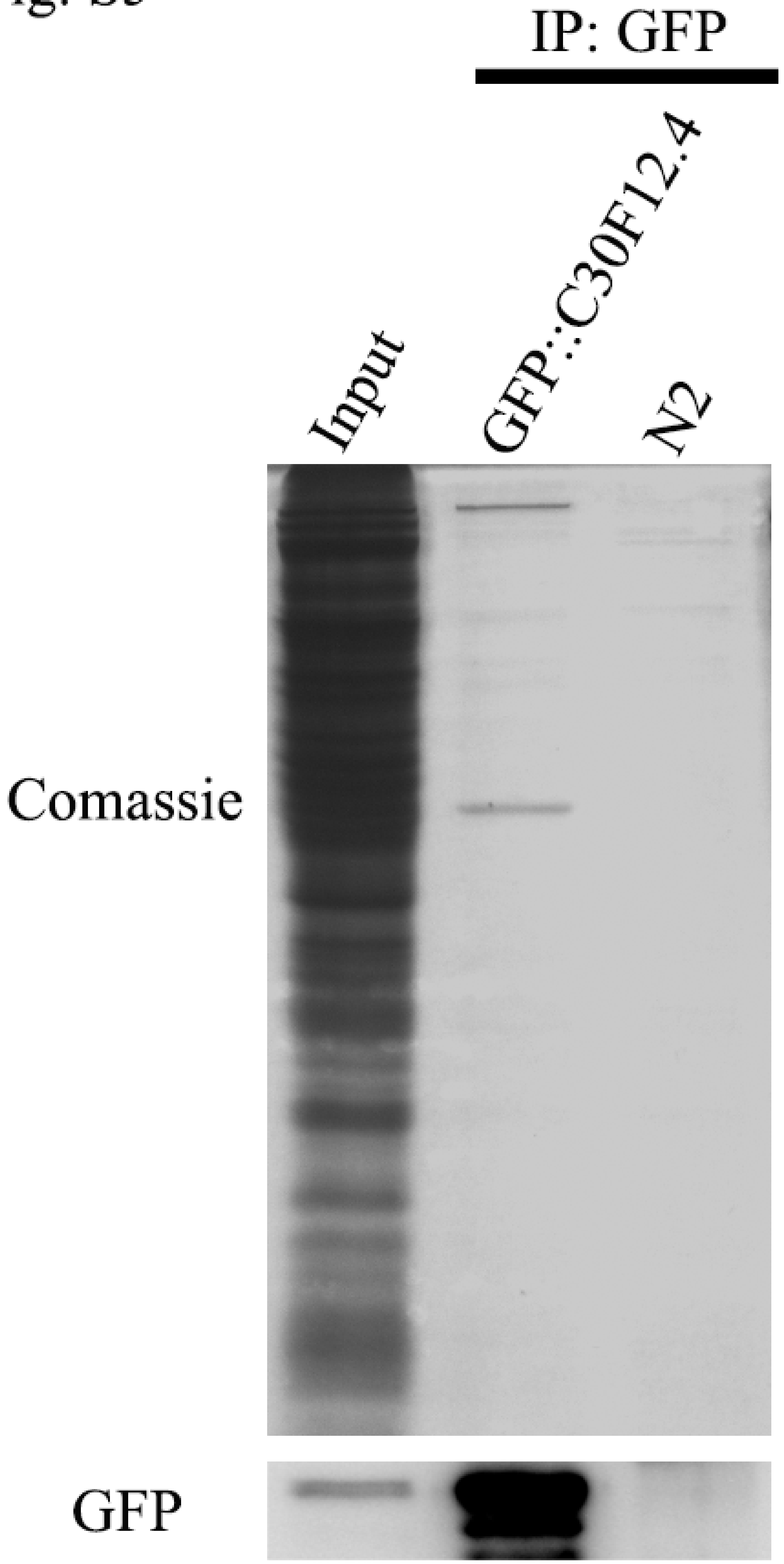

Fig. S6

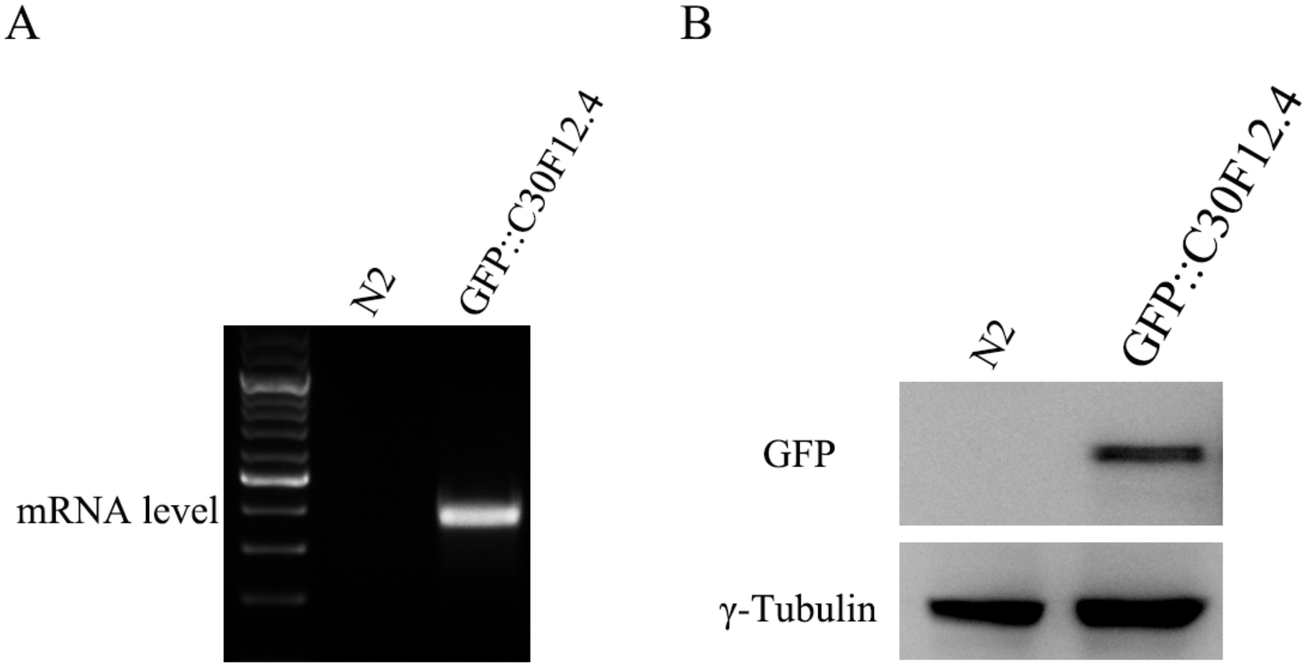

Supplement: Supplementary file 1 — Supplementary material 1 (PDF 986 kb) [file 13238_2016_308_MOESM1_ESM.pdf]
